# The Possibility of Influencing the Malignant Neoplasmata by Inoculation

**Published:** 1910-06-04

**Authors:** G. S. Thompson


### SPECIAL ARTICLE.

### THE POSSIBILITY OF INFLUENCING THE MALIGNANT NEOPLASMATA BY INOCULATION.

By G. S. THOMPSON, M.R.C.S., L.R.C.P.

As I am unaware what, if any, investigations have been carried out on the subject with which this communication deals, I submit the following suggestion for what it is worth to those who may have the time, will, and means of putting the matter to the test. It appears to me that the idea about to be developed has sufficient experimental and theoretical basis to merit trial and to be worthy of research. The subject with which I propose to deal is that of neoplasmomic inoculation as a therapeutic remedy in the treatment of malignant growths.

I think we all realise that the present method of surgical treatment by ablation is unsatisfactory and can be only temporary, although it is the most satisfactory in our present stage of therapeutic evolution. At the most it is a crude and coarse procedure, which, for want of a better, we are perforce obliged to follow. That it is unsatisfactory, however, will not, I think, be disputed. Its sphere of usefulness is limited by time and position, and the mutilation in act, appearance, and function little commends itself to our æsthetic and scientific sense, whilst its sphere of usefulness is often limited by anatomical or vital considerations. The frequent difficulty of interference or recognition owing to anatomical barriers and inaccessible positions till unmistakable signs indicate the passage beyond the stage of practical or prudent surgery; the dangers, difficulties, complications, immediate and remote mortality; the physical and mental anguish as well as the parlous condition in the later stages: these, to mention only a few, are but some of the drawbacks which stimulate our desire and effort to the discovery of a more certain, satisfactory and gentler remedy.

Naturally one comes forward with diffidence in this province where so many able and thorough investigators have failed, and this feeling has restrained me bringing the matter forward before; but in the hope that there may be something of value I think it only right to offer these indifferent remarks for what they are worth.

Presumably the time will come when we shall try —following the indications of Nature—to influence malignant tumours by exploiting the hidden mysteries and wonderful properties of the blood, thus effectually and adequately overcoming the present obstacles and difficulties and realising our hope of vanquishing the offender *in situ*.

The following idea first occurred to me during a lecture, delivered by Sir A. E. Wright, on the subject of immunity dealing in particular with the elaboration of anti-bodies in the blood in response to inoculations. Thus we have (to use Wright's term) the various tropic anti-bacterial substances as agglutinins, opsonins, bactericidal and bacteriolytic bodies: the anti-cytotropic bodies in response to inoculations of parenchymatous protoplasm, such as erythrocytotropic and hæmolytic substances (immune bodies and complements), leucocytotropic, spermatocytotropic, neurocytotropic, nephrocytotropic, hepatocytotropic, tracheocytotropic substances and so on, causing cytoparalysis and cytolysis of red and white corpuscles, spermatozoa, nerve cells, kidney, hepatic and tracheal cells respectively: the anti-albuminoid bodies as the precipitins, anti-rennins, anti-coagulins, anti-complements, anti-ferments: the phytotoxotropic substances as anti-ricin and anti-abrin. These phenomena, so far as this particular aspect of hæmatology has been investigated, appear to be special instances of a general law to the effect that the inoculation into the body of proteous or proteoid substances induces the elaboration of anti-bodies in response thereto, the object and effect of which is the antagonisation of the invading material, not only sufficiently for this purpose but also in excess of this necessity. Thus we find that inoculation of emulsion of liver tissue induces the elaboration of anti-hepatic substances in the blood, the excess of which over that necessary to antagonise the injected material attacking the liver cells of the organ itself, producing patches of focal necrosis therein: and similarly for the other organs, a like effect applying to all the tissues.

Now it appeared to me that inoculation of a growth emulsion would provoke an elaboration of anti-neocytotropic substances resulting in opsonisation, neutralisation, and necrosis of the mycelia, followed by cytolysis or phagocytosis—in fact, that the law of negative and positive variation of neocytolysins would here, as in other instances, become operative. Assuming, therefore, that in an affected patient the neocytolytic index were subnormal, by carefully adjusted and properly timed inoculations an endeavour would be made to raise the neocytolytic index by positive phasal cumulation, and thereafter maintain the same at a constant high level. This richly cytolytic serum of necessity coming into intimate relation with the lesions, or being "determined" there at every portal of blood supply, would exercise its specific antagonising, destructive, and eliminating influence. Perhaps such or a like process operates in the well-recognised instances of malignant arrest, involution or dispersal.

That the body has some varying power of dealing with cancer would appear highly probable, if not certain. How and what this is, is the question an answer to which will give the true and seemingly most satisfactory solution to a remedy. On evolutionary grounds this assumption is, to say the least, extremely probable, for would it not seem strange and extraordinary that natural selection should have evolved a protective armoury against most if not all other pathogenic intruders, to be found alone wanting here? Such an idea would be in violent conflict with certain monistic hypotheses with their insensitive gradationary principles. Our hard and fast ideas as to malignancy must give way to the universal growth law of the gradation of evolutionary and involutionary sequency. One is strongly inclined to think that no rigid distinction of benign and malignant as applied to tumours holds as understood at the present day, but that there is a gradation of change from one extreme to the other; that the benign growths are not sharply defined from the malignant, but connected by a transition of continuous uninterrupted gradations; that the difference is not one of kind, but rather degree; and that possibly under changes of resistance or other conditions with which we are not well acquainted the passage from one state to the other may actually be realised in the same growth, and *vice versâ*.

The facts in favour of, and that tend to give colour to, these views are the varying phases of progression and inactivity in malignant growths, their spontaneous arrest and partial or complete involution, as well as their prolonged latency. Instances illustrating these statements have been, and continue to be, recorded frequently by thoroughly competent writers where such conditions as chronic inflammatory disorders, post-gummatous sclerosis of liver simulating cancer, etc., have, of course, been eliminated—observations based on clinical pathological and microscopic findings that leave no doubt as to their accuracy and correctness. Malignant transformation of simple tumours is comparatively common. Certain adenomata and growths, as myelomata, appear to occupy an intermediate or connecting position. The apparent benignancy or slight malignancy of the myelomata, where possibly the myeloplaxes exercise a restraining action, suggests a natural effort of resistance. There are many cases on record now in which the unquestionable malignancy of spontaneously dispersed growths leaves no doubt as to the ability of natural processes successfully to cope with these tumours. In fact, it seems certain that the organism has a varying power of neutralising and vanquishing one of its powerful pathological enemies (if malignant disease may be so regarded). If this be true, and I think there can be little doubt on the point, it should, and must, be our endeavour to unravel and discover the processes at work. To then follow up, exploit, and intensify these phenomena would appear to be the method giving most promise of a successful, gratifying, and satisfactory issue to our labours.

I have already indicated a possible means whereby this consummation may be attained by neoplasmomic inoculation, that is to say, inoculation into the individual concerned of a suitably prepared and adjusted malignant tissue emulsion or the active principles thereof. Whether the cell emulsion would have to be prepared from the particular growth sought to be resolved, or from a similar or different variety of the sub-group of malignant tumours, could only be ascertained by trial. The emulsion must, of course, be sterile, and so maintained. Experimental test inoculation would establish the matter of dosage, for it is conceivable that an excessive dose of the protoplasm might, amongst other things, cause fatal thrombosis. Furthermore, the cells would require spontaneous or artificial devitalisation (but not destruction), so as to obviate possible growth at the site of inoculation. The difficulty of supply might be overcome by obtaining material from growths produced elsewhere by transplantation or inoculation, and a passive, as opposed to an active, immunity could possibly be effected by the use of immunised serum, whilst some method of auto-inoculation might be evolved and used in certain cases not amenable to the other methods, etc., etc.

There is scarcely anything I can offer as practical demonstration in favour of this hypothesis, but this is due rather to lack of opportunity, means, and other difficulties than want of desire to undertake the research. One case, however, in which the above principle was roughly applied suggests, in harmony with other theoretical considerations, the hope that this line of treatment, when developed, might be crowned with an unhoped-for success. The patient referred to was a man, with recurrent sarcoma of the maxilla, introduced at a medical society's meeting, with requests for suggestions as to treatment. The novel suggestion I made was, to my surprise, adopted by the surgeon in charge. Some of the growth excised under aseptic and antiseptic precautions was placed in a sterile test tube for three days, so as to ensure cell necrosis. Thereafter a second anæsthetic was administered and the graft implanted under an incision in the abdominal wall. Unfortunately, being absent from the first operation, I subsequently learnt that only one piece of growth was excised, thus limiting ensuing implantations to one operation. Owing to difficulties of preparation of an emulsion we were compelled to use the small piece of growth *en masse*. At the time of operation the growth, by its size, had caused closure of the palpebral fissure, nasal obstruction, and depression of the hard palate on the same side. The implantation wound healed by first intention, and at the end of three weeks the patient left hospital, but did not return. Towards the end of his time there appeared to be noticeable diminution in size of the growth, but more striking than this was the unsolicited statement of the patient that he could again partly see out of the affected eye.

Whether the foregoing surmises have or have not any intrinsic value, I feel sure that the right method of trying to solve the puzzle of cancer treatment is by focusing our attention and efforts fundamentally on the blood pathology of malignancy, to endeavour to unravel in what way Nature undoubtedly acts beneficially, and sometimes even successfully, and then to reproduce, augment, and give effective incidence to these powers by suitable means. I commend these ideas to the consideration of those more able than myself to give them a practical application. They are not the outcome of any research, but merely suggestions arising from inductive reasoning based on certain biological facts. To elaborate and control carcinomato- and sarcomato-cytotropic substances on the one hand and give them the most effective local onslaught on the other is the alpha and omega of treatment, a problem the solution of which may terminate the tyrannical sway of our well-nigh invulnerable foe. "The blood is the life."

